# Supplementary figures and images for: Pumilio differentially binds to mRNA 3′ UTR isoforms to regulate localization of synaptic proteins
Source: EMBO Rep. 2025 Feb 21;26(7):1792–815. doi: 10.1038/s44319-025-00401-z (PMC11976915; doi:10.1038/s44319-025-00401-z)

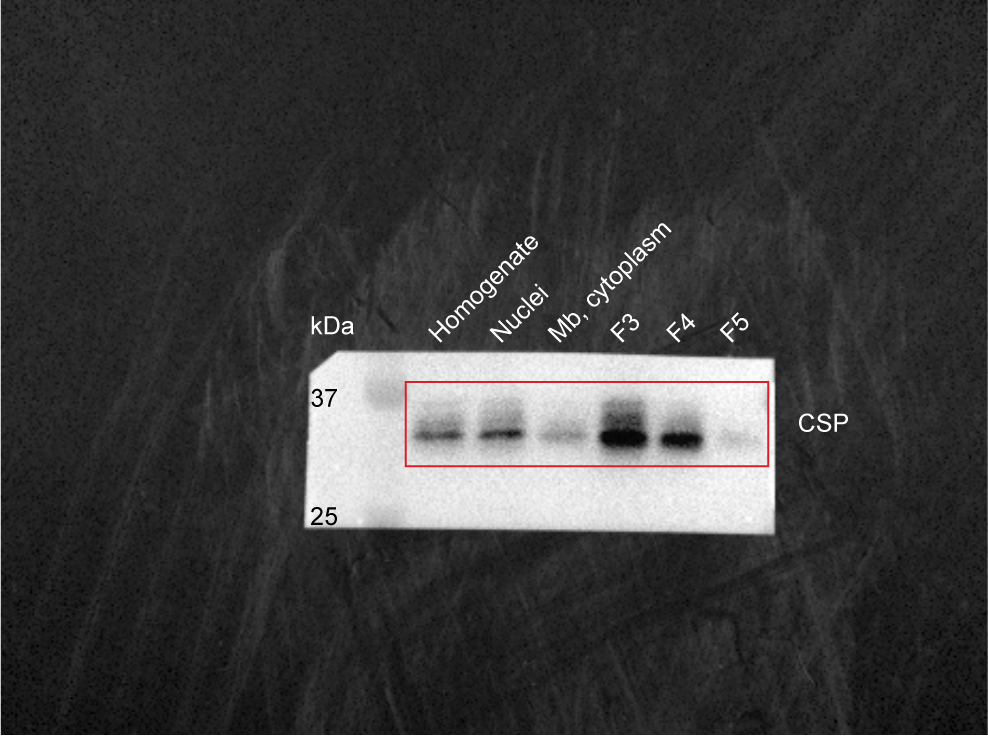

Supplement: Supplementary file 7 — Source data Fig. 2 [file 44319_2025_401_MOESM7_ESM.zip › EMBOR-2024-60013V2_SourceDataForFigure2/2C/CSP_western_annotated.tif]

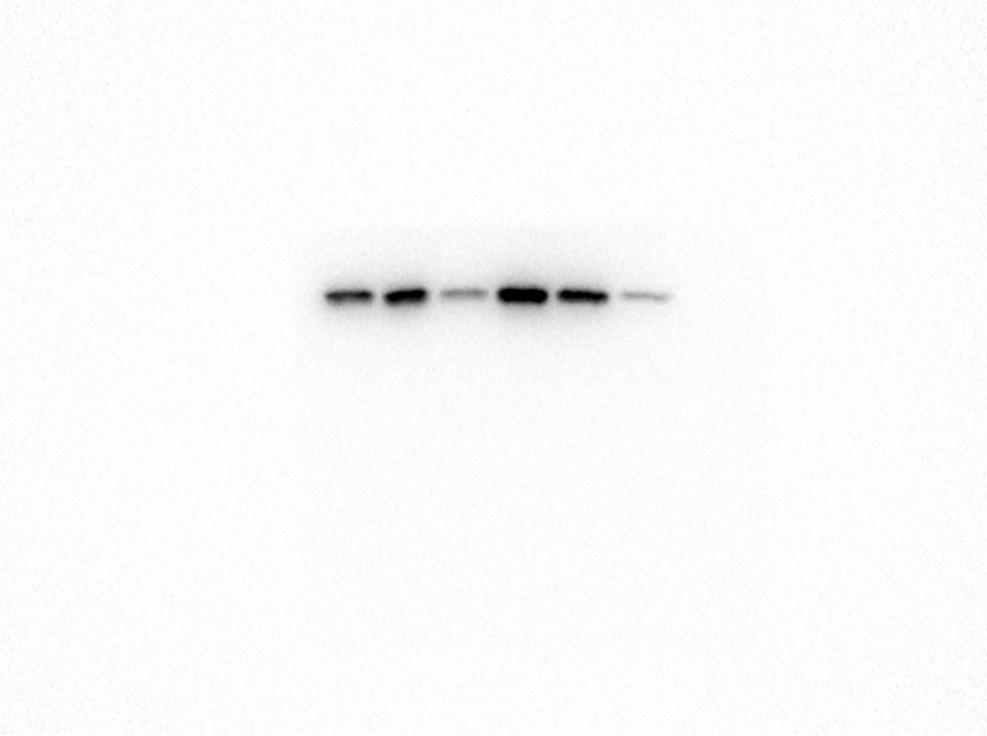

Supplement: Supplementary file 7 — Source data Fig. 2 [file 44319_2025_401_MOESM7_ESM.zip › EMBOR-2024-60013V2_SourceDataForFigure2/2C/Syx1A_western.tif]

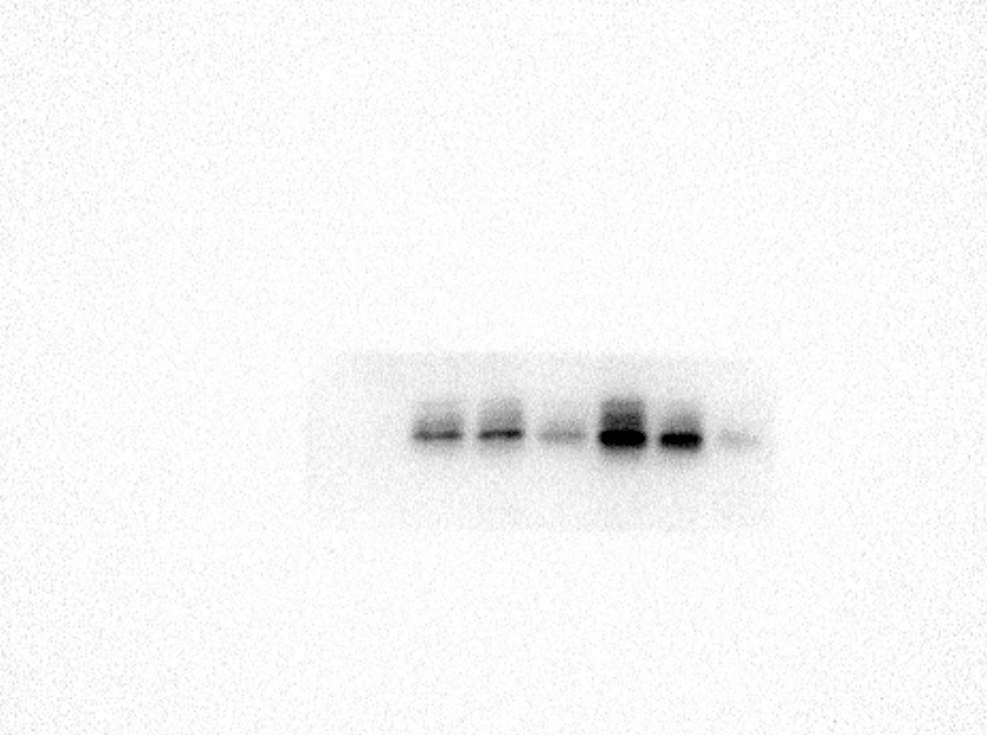

Supplement: Supplementary file 7 — Source data Fig. 2 [file 44319_2025_401_MOESM7_ESM.zip › EMBOR-2024-60013V2_SourceDataForFigure2/2C/CSP_western.tif]

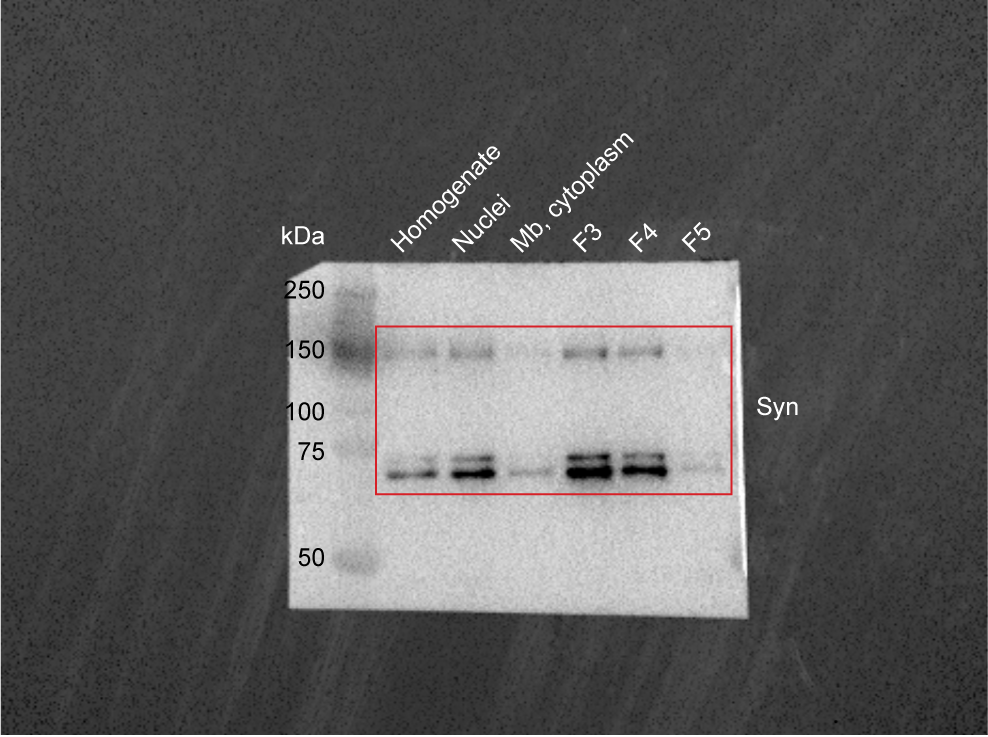

Supplement: Supplementary file 7 — Source data Fig. 2 [file 44319_2025_401_MOESM7_ESM.zip › EMBOR-2024-60013V2_SourceDataForFigure2/2C/Syn_western_annotated.tif]

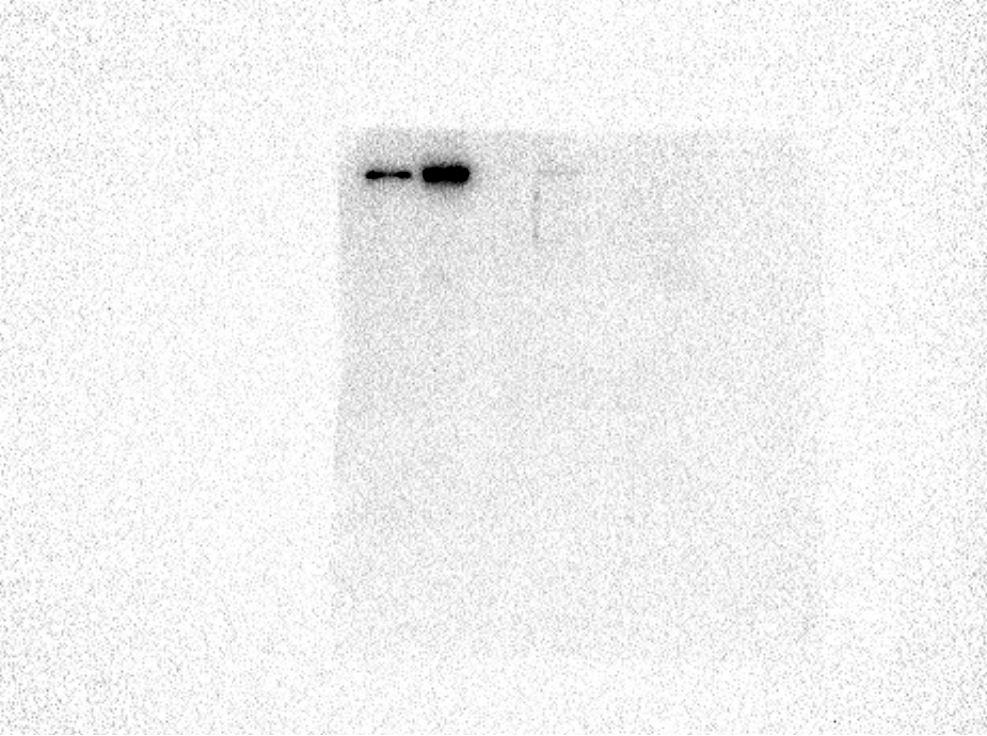

Supplement: Supplementary file 7 — Source data Fig. 2 [file 44319_2025_401_MOESM7_ESM.zip › EMBOR-2024-60013V2_SourceDataForFigure2/2C/LaminC_western.tif]

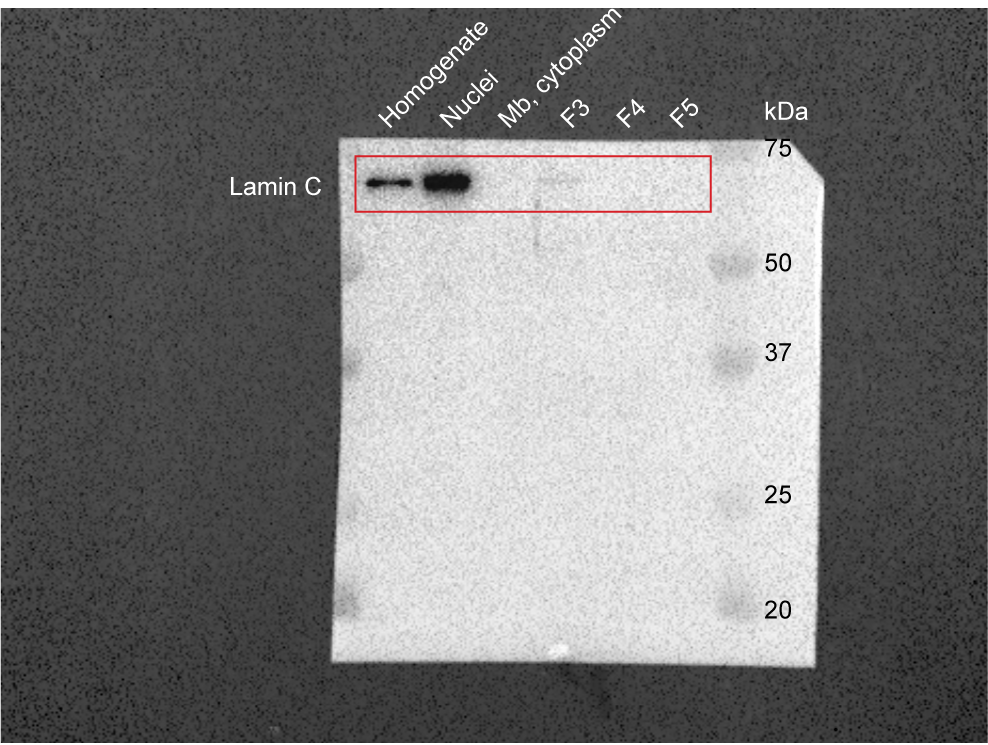

Supplement: Supplementary file 7 — Source data Fig. 2 [file 44319_2025_401_MOESM7_ESM.zip › EMBOR-2024-60013V2_SourceDataForFigure2/2C/LaminC_western_annotated.tif]

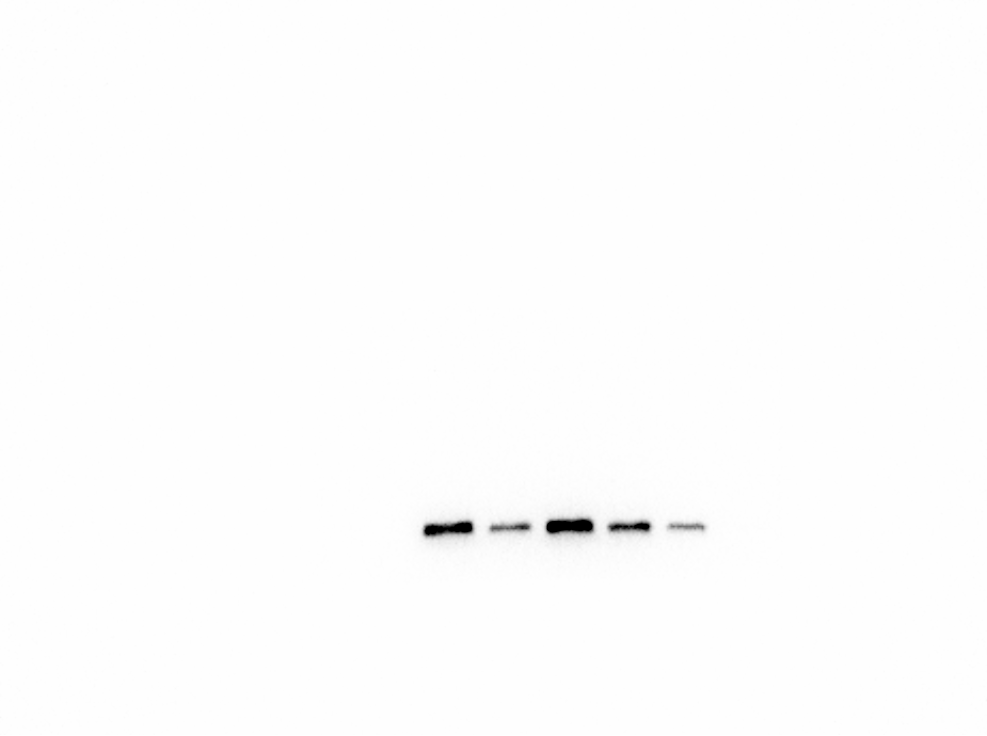

Supplement: Supplementary file 7 — Source data Fig. 2 [file 44319_2025_401_MOESM7_ESM.zip › EMBOR-2024-60013V2_SourceDataForFigure2/2C/alphaTubulin_western.tif]

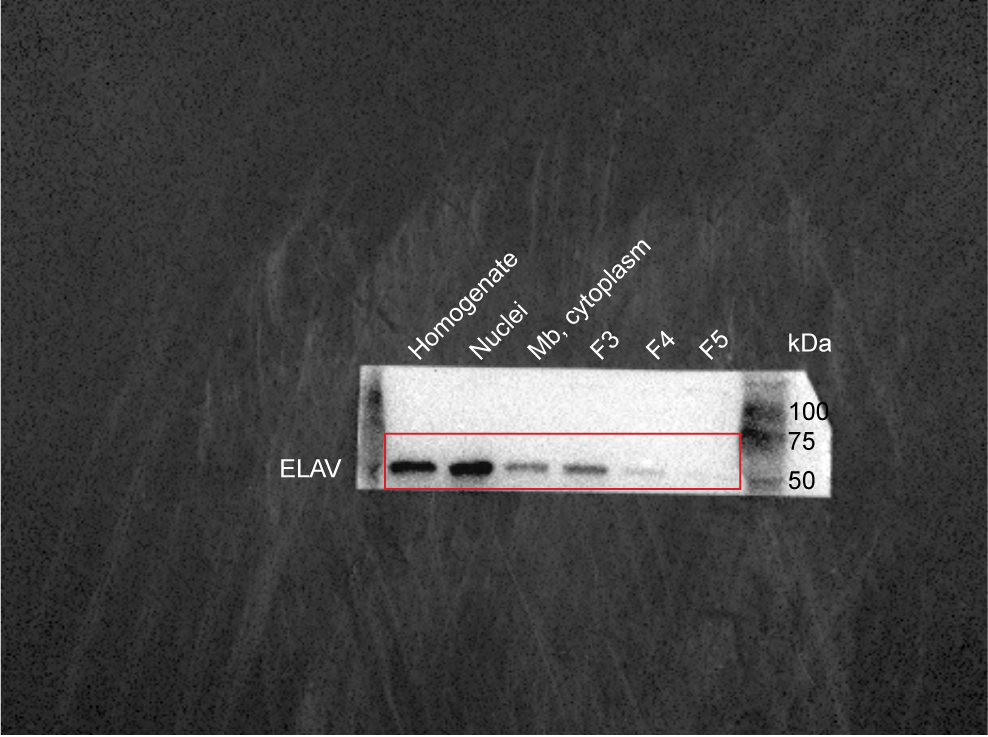

Supplement: Supplementary file 7 — Source data Fig. 2 [file 44319_2025_401_MOESM7_ESM.zip › EMBOR-2024-60013V2_SourceDataForFigure2/2C/ELAV_western_annotated.tif]

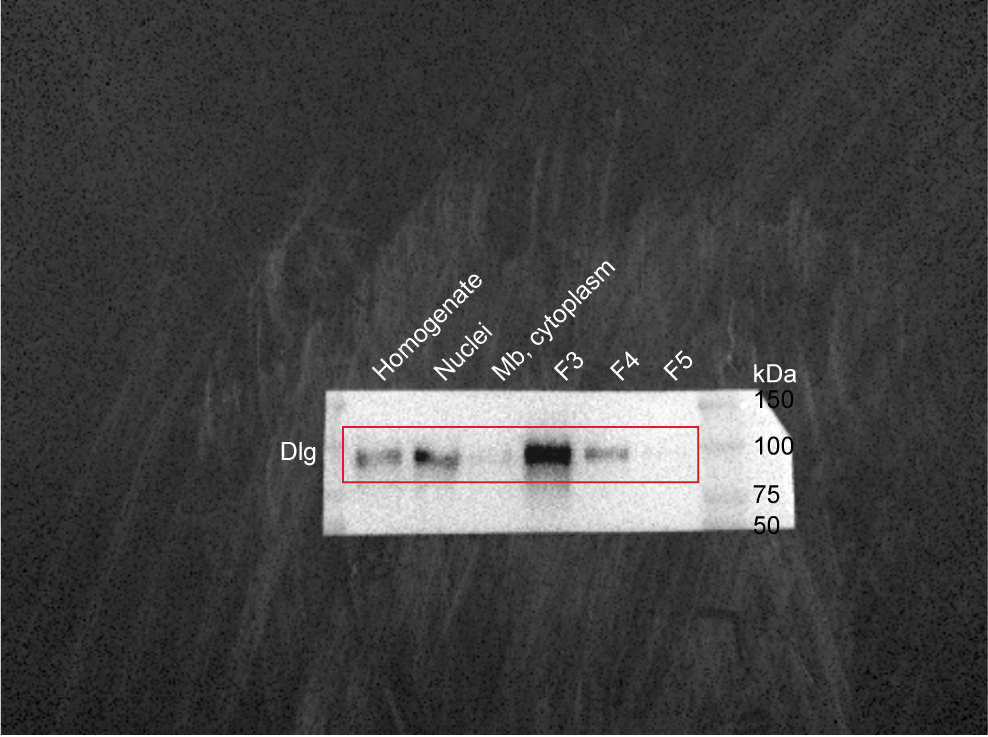

Supplement: Supplementary file 7 — Source data Fig. 2 [file 44319_2025_401_MOESM7_ESM.zip › EMBOR-2024-60013V2_SourceDataForFigure2/2C/Dlg_western_annotated.tif]

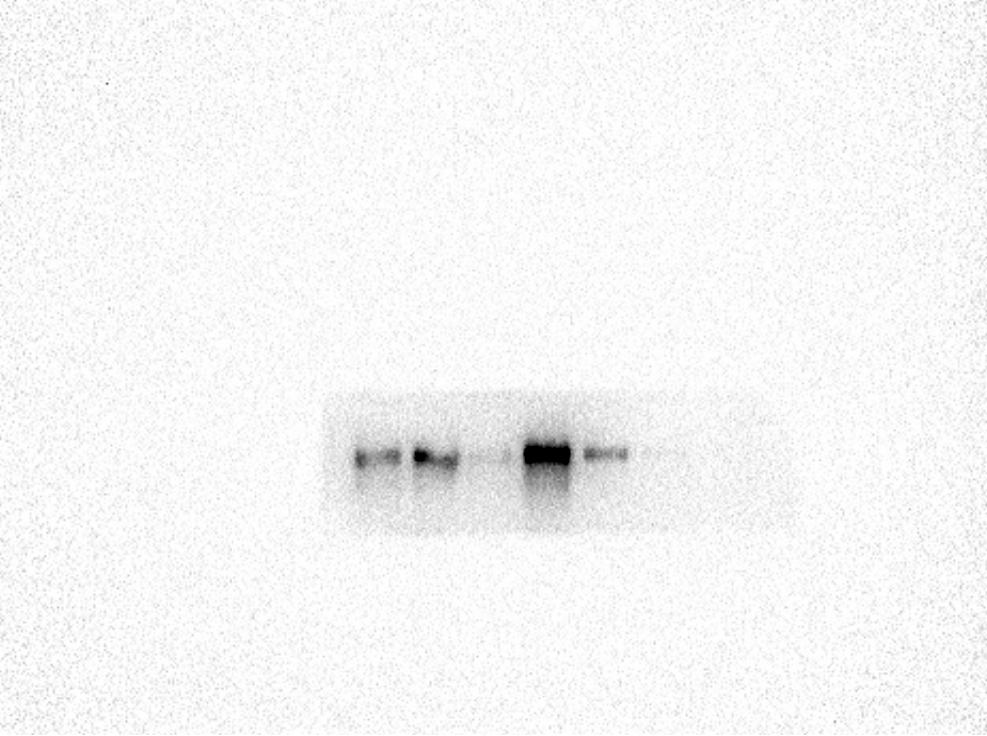

Supplement: Supplementary file 7 — Source data Fig. 2 [file 44319_2025_401_MOESM7_ESM.zip › EMBOR-2024-60013V2_SourceDataForFigure2/2C/Dlg_western.tif]

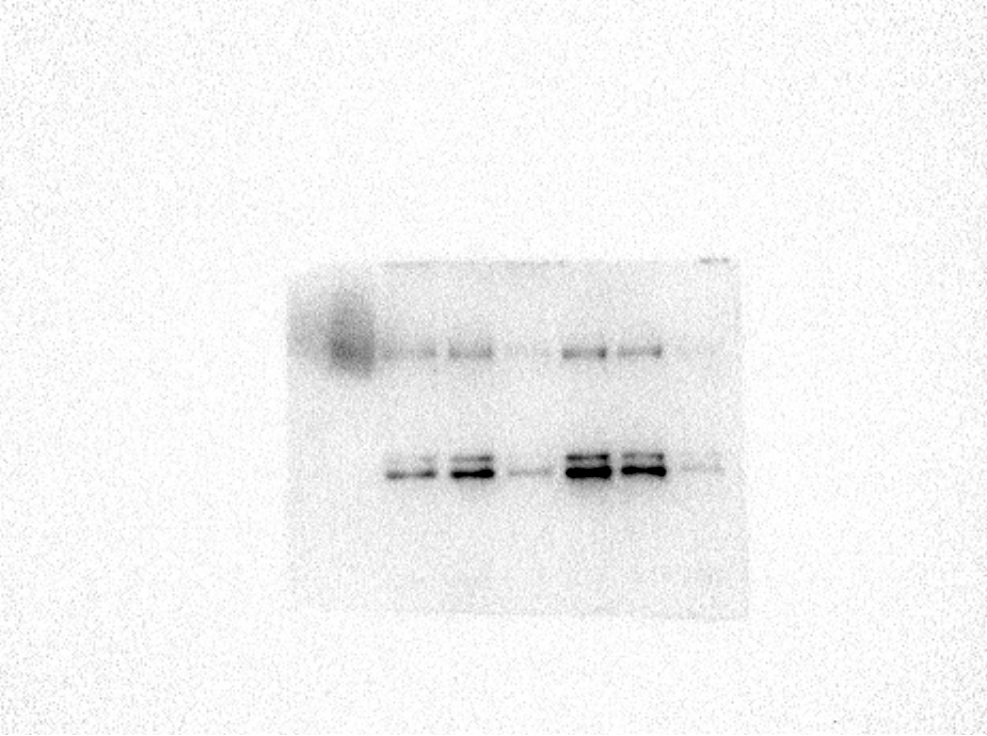

Supplement: Supplementary file 7 — Source data Fig. 2 [file 44319_2025_401_MOESM7_ESM.zip › EMBOR-2024-60013V2_SourceDataForFigure2/2C/Syn_western.tif]

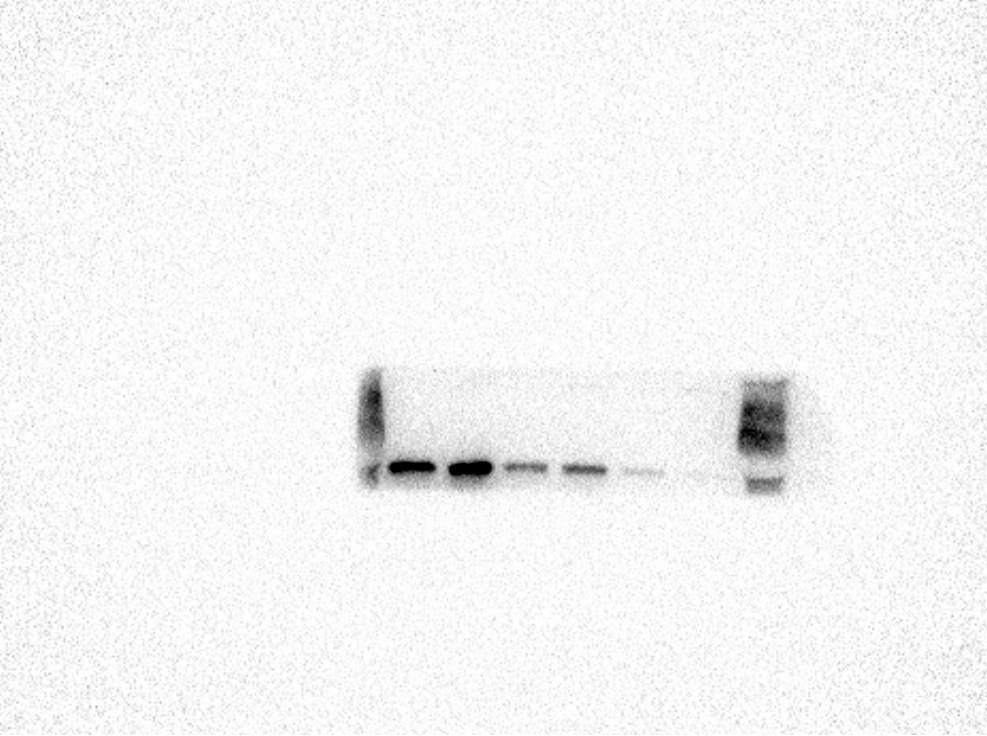

Supplement: Supplementary file 7 — Source data Fig. 2 [file 44319_2025_401_MOESM7_ESM.zip › EMBOR-2024-60013V2_SourceDataForFigure2/2C/ELAV_western.tif]

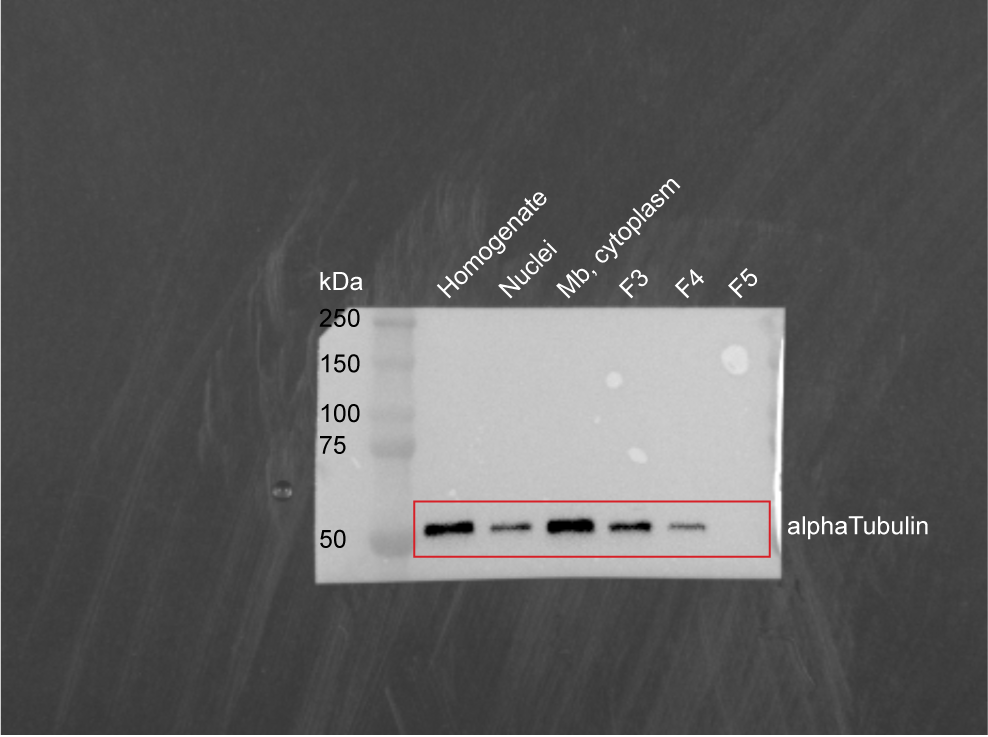

Supplement: Supplementary file 7 — Source data Fig. 2 [file 44319_2025_401_MOESM7_ESM.zip › EMBOR-2024-60013V2_SourceDataForFigure2/2C/alphaTubulin_western_annotated.tif]

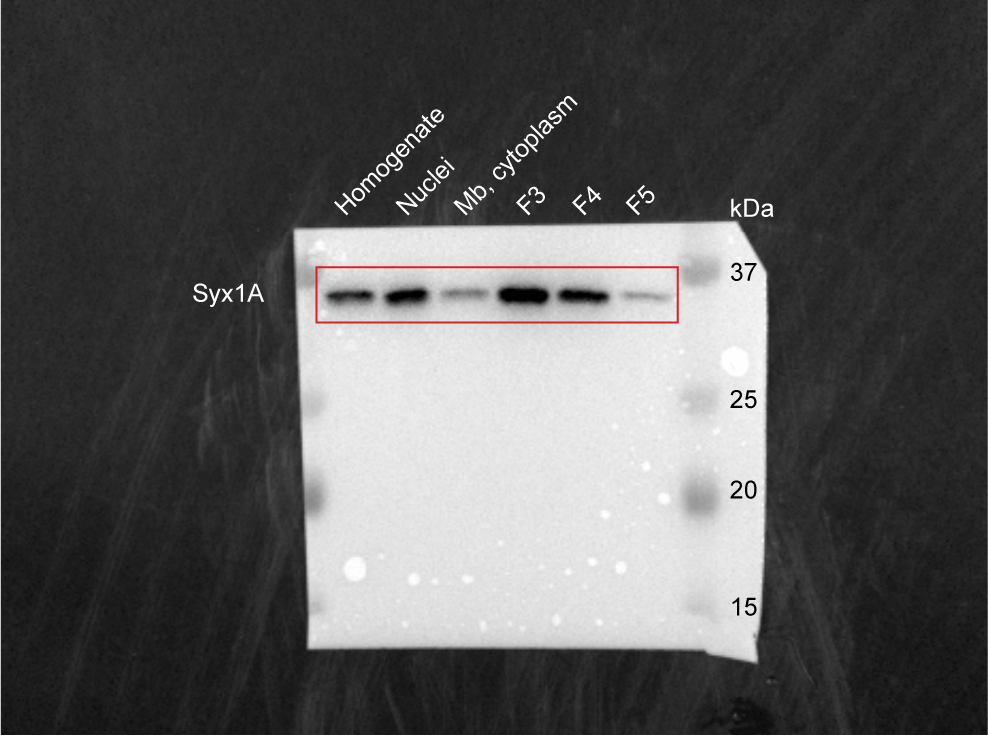

Supplement: Supplementary file 7 — Source data Fig. 2 [file 44319_2025_401_MOESM7_ESM.zip › EMBOR-2024-60013V2_SourceDataForFigure2/2C/Syx1A_western_annotated.tif]

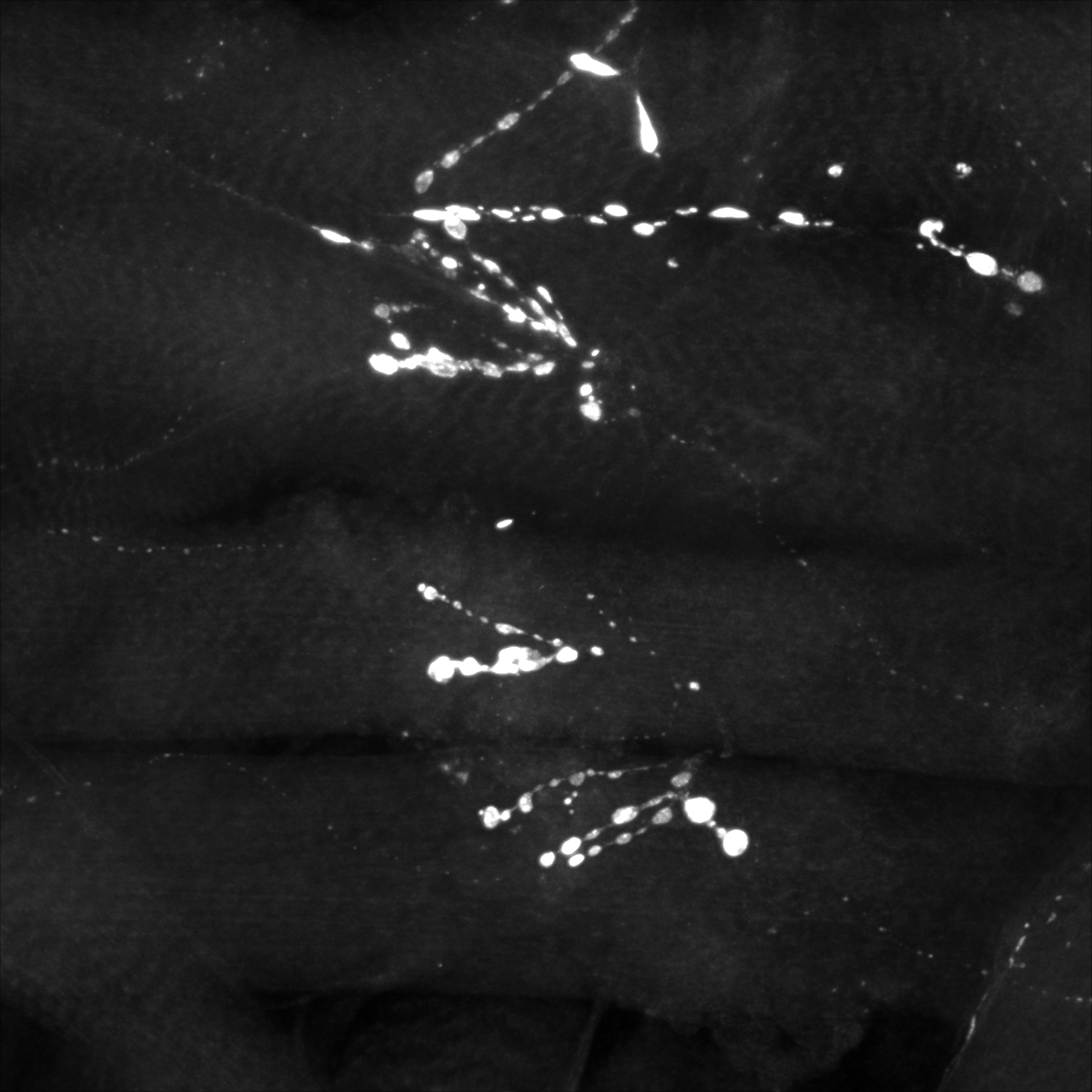

Supplement: Supplementary file 8 — Source data Fig. 5 [file 44319_2025_401_MOESM8_ESM.zip › EMBOR-2024-60013V2_SourceDataForFigure5/5E/Pum_KO_Syt1_max_intensity_projection.tif]
