# Supplementary material for: Pumilio differentially binds to mRNA 3′ UTR isoforms to regulate localization of synaptic proteins
Source: EMBO Rep. 2025 Feb 21;26(7):1792–815. doi: 10.1038/s44319-025-00401-z (PMC11976915; doi:10.1038/s44319-025-00401-z)
Supplement: Supplementary file 9 — Expanded View Figures [file 44319_2025_401_MOESM9_ESM.pdf]

Expanded View Figures

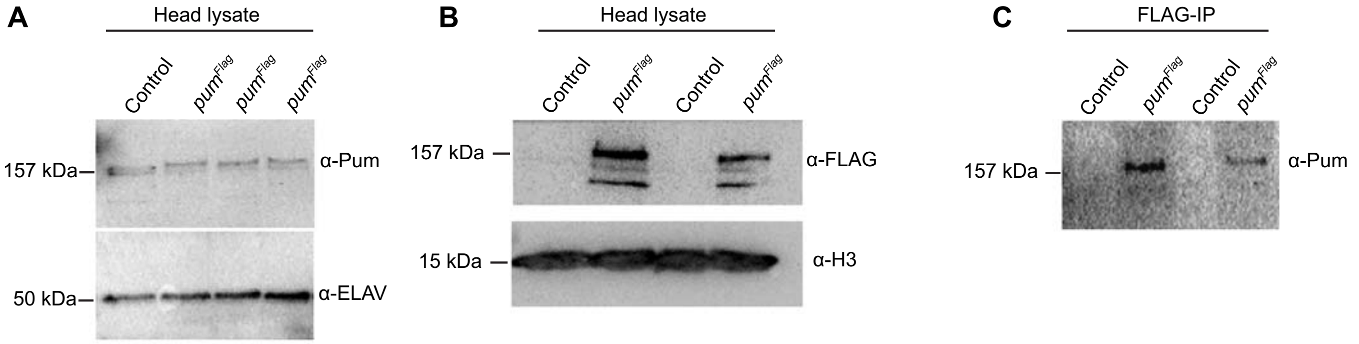

D

Motif	RNA-binding protein	E-value	% occurrence in Pum targets	% occurrence in background
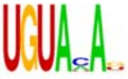	PUM	1.14e-43	74.1	38.0
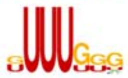	ORB2	1.29e-20	79.0	53.8
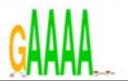	PABP	2.26e-19	71.7	46.5
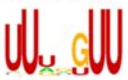	RBP9	1.38e-17	82.8	60.1
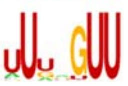	FNE	2.57e-16	75.2	52.0

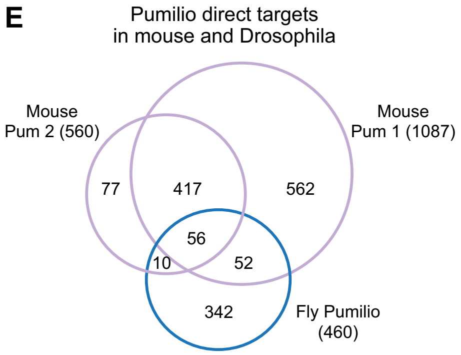

Figure EV1. xRIP-3′-seq identifies mRNAs directly bound by Drosophila Pumilio.

(A–C) Western blots showing Pum expression in adult fly heads. (A) Detection with an anti-Pum antibody shows the molecular weight difference between wild-type Pum (untagged) compared to Flag-HA-tagged Pum protein in three independent *pum*^{Flag} transformant flies. ELAV serves as loading control. (B) Detection with an anti-Flag antibody shows specific detection of Pum-Flag in *pum*^{Flag} flies. Histone H3 serves as a loading control. Five fly heads were used for protein preparation for each genotype. (C) Eluates from an anti-Flag antibody immunopurification of Pum from head extract of *pum*^{Flag} and control (w^{1118}) flies. (D) The Pumilio Response Element (PRE) constitutes the most enriched motif in the 3′ UTRs of Pum target mRNAs ($p = 1.14*10^{-43}$; E-value). Motifs for the top five RBPs are shown. (E) Venn diagram showing the intersection between Pum mRNA targets in Drosophila (this study) and for mammalian Pum 1 and Pum 2 identified in Zhang et al, 2017 (Zhang et al, 2017).

© The Author(s)

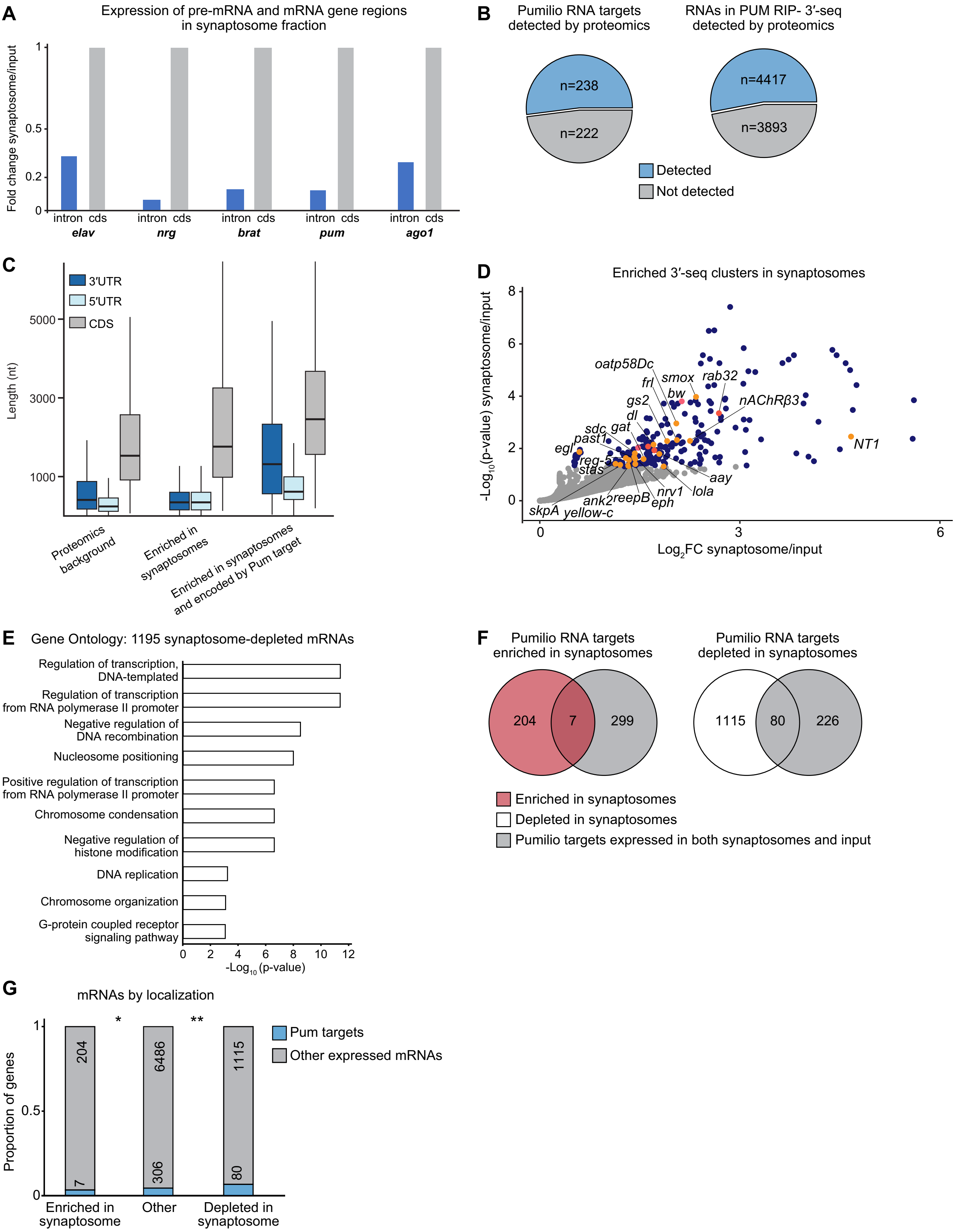
A
Expression of pre-mRNA and mRNA gene regions in synaptosome fraction
Fold change synaptosome/input
intron cds
elav
nrg
brat
pum
ago1
B
Pumilio RNA targets detected by proteomics
RNAs in PUM RIP- 3′-seq detected by proteomics
n=238
n=222
n=4417
n=3893
Detected
Not detected
C
3′UTR
5′UTR
CDS
Length (nt)
Proteomics background
Enriched in synaptosomes
Enriched in synaptosomes and encoded by Pum target
D
Enriched 3′-seq clusters in synaptosomes
-Log10(p-value) synaptosome/input
Log2FC synaptosome/input
oatp58Dc
frl
smox
rab32
bw
gs2
nAChRβ3
dl
sdc
gat
past1
egl
reg-5
stas
NT1
aay
lola
nrv1
ank2
reepB
eph
skpA
yellow-c
E
Gene Ontology: 1195 synaptosome-depleted mRNAs
Regulation of transcription, DNA-templated
Regulation of transcription from RNA polymerase II promoter
Negative regulation of DNA recombination
Nucleosome positioning
Positive regulation of transcription from RNA polymerase II promoter
Chromosome condensation
Negative regulation of histone modification
DNA replication
Chromosome organization
G-protein coupled receptor signaling pathway
-Log10 (p-value)
F
Pumilio RNA targets enriched in synaptosomes
Pumilio RNA targets depleted in synaptosomes
204
7
299
1115
80
226
Enriched in synaptosomes
Depleted in synaptosomes
Pumilio targets expressed in both synaptosomes and input
G
mRNAs by localization
Proportion of genes
204
7
6486
306
1115
80
*
**
Pum targets
Other expressed mRNAs
Enriched in synaptosome
Other
Depleted in synaptosome

© The Author(s)

Figure EV2. Pum target mRNAs encode proteins enriched in synaptosome fractions.

(**A**) RT-qPCR quantification of the indicated transcript regions in synaptosome fractions relative to input fraction. For each gene, intron levels were normalized to coding exon mRNA levels, which were set to the value 1. Ratios represent the average of two biological replicates. (**B**) Proportions of proteins encoded by Pum target mRNAs (left) and all RNAs identified in Pum xRIP-3′-seq experiment (right) that were detected by proteomics in the synaptosome isolation experiment. (**C**) Average length of CDS, 5′ UTR and 3′ UTR, of mRNAs encoding proteins of the indicated categories. Number of genes analyzed: $n = 4843$ (Proteomics background), $n = 989$ (enriched in synaptosomes) and $n = 116$ (enriched in synaptosomes and encoded by a Pum target gene). Boxes indicate range between minimum and maximum, the central line depicts the median, lower and upper bounds represent the first and third quartiles, respectively. (**D**) Differential mRNA expression (by 3′-seq cluster expression) in synaptosome fractions compared to input. The *p*-value of the enrichment is represented as a function of $\log_2$ fold change. Dark blue represents $|\log_2$ fold change (synaptosome/input)$|$ >0 and p-value < 0.05 (Wald test). (**E**) Gene ontology analysis of 1195 mRNAs depleted in synaptosome fractions compared to input. The top ten terms are shown ($p < 0.01$; one-sided EASE score adjusted using the Benjamini-Hochberg method). See Dataset EV3 for all significant terms. (**F**) Venn diagram showing the intersection between Pum target mRNAs and mRNAs enriched (left) or depleted (right) in the synaptosome fraction. (**G**) Number and proportion of Pum target mRNAs in each category of mRNA subcellular localization. *$p = 0.1$, **$p < 0.05$ ($p = 4e{-9}$) (two-tailed Fisher's exact test). Only Pum target mRNAs expressed in both synaptosome and input (306 genes) were considered.

 © The Author(s)

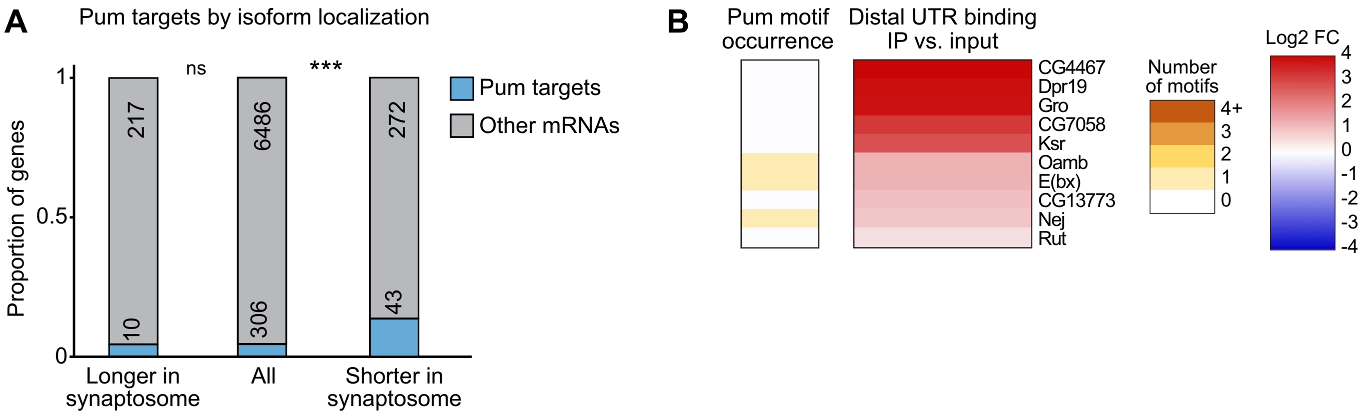

Figure EV3. Synaptic localization of short 3′ UTR isoforms of Pumilio target mRNAs.

(**A**) Number and proportion of Pum target genes in each category of 3′ UTR isoform subcellular localization. ns, non-significant, ***$p < 0.001$ ($p = 4e{-}10$, two-tailed Fisher's exact test). Only Pum target mRNAs expressed in both synaptosome and input (306 genes) were considered. (**B**) Heatmaps showing Pum binding and the number of Pum binding motifs in distal 3′ UTR regions of Pum target mRNAs that display longer 3′ UTR isoforms in synaptosomes (10 genes), ranked by Pum xRIP-3′-seq signal compared to input.

© The Author(s)

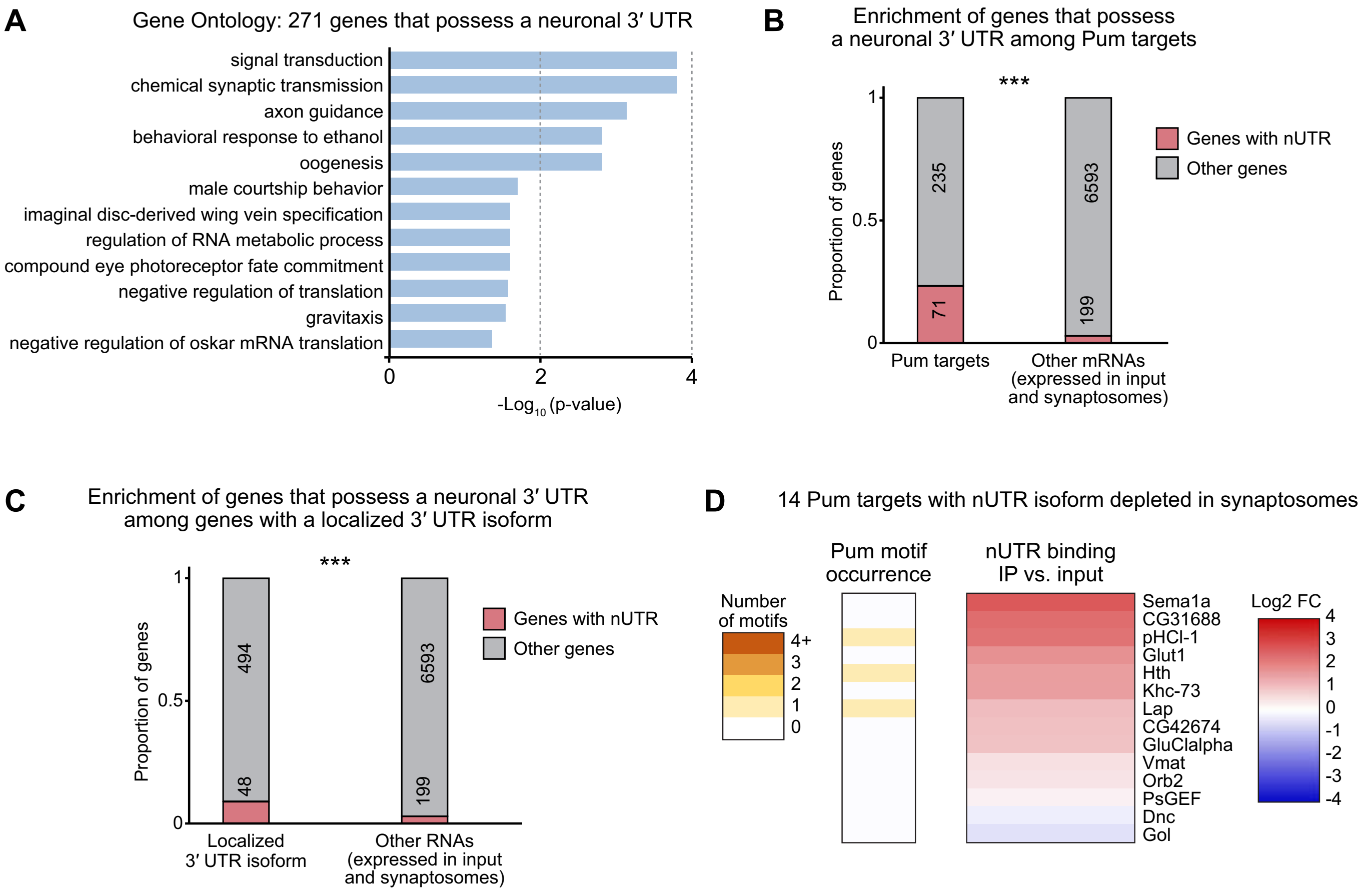

Figure EV4. Pumilio binds to soma-localized long neuronal 3′ UTRs of synaptic genes.

(**A**) Gene ontology analysis of 271 genes that possess a neuronal 3′ UTR. All significant terms are shown ($p < 0.05$, one-sided EASE score adjusted using the Benjamini-Hochberg method). See also Dataset EV4. (**B**) Number and proportion of nUTR-containing genes in each gene category. ***$p < 0.001$ ($p = 2e{-}44$, two-tailed Fisher's exact test). Only mRNAs expressed in both the synaptosome and input samples (199 nUTR-containing genes) were considered. (**C**) Number and proportion of nUTR-containing genes in each category of 3′ UTR isoform subcellular localization. ***$p < 0.001$ ($p = 2e{-}10$, two-tailed Fisher's exact test). Only mRNAs expressed in both the synaptosome and input samples (199 nUTR-containing genes) were considered. (**D**) Heatmaps showing Pum binding and the number of Pum binding motifs in the nUTR of Pum target mRNAs in genes whose nUTR-containing 3′ UTR isoform is depleted in synaptosome fractions (14 genes), ranked by Pum xRIP-3′-seq signal compared to input.

 © The Author(s)

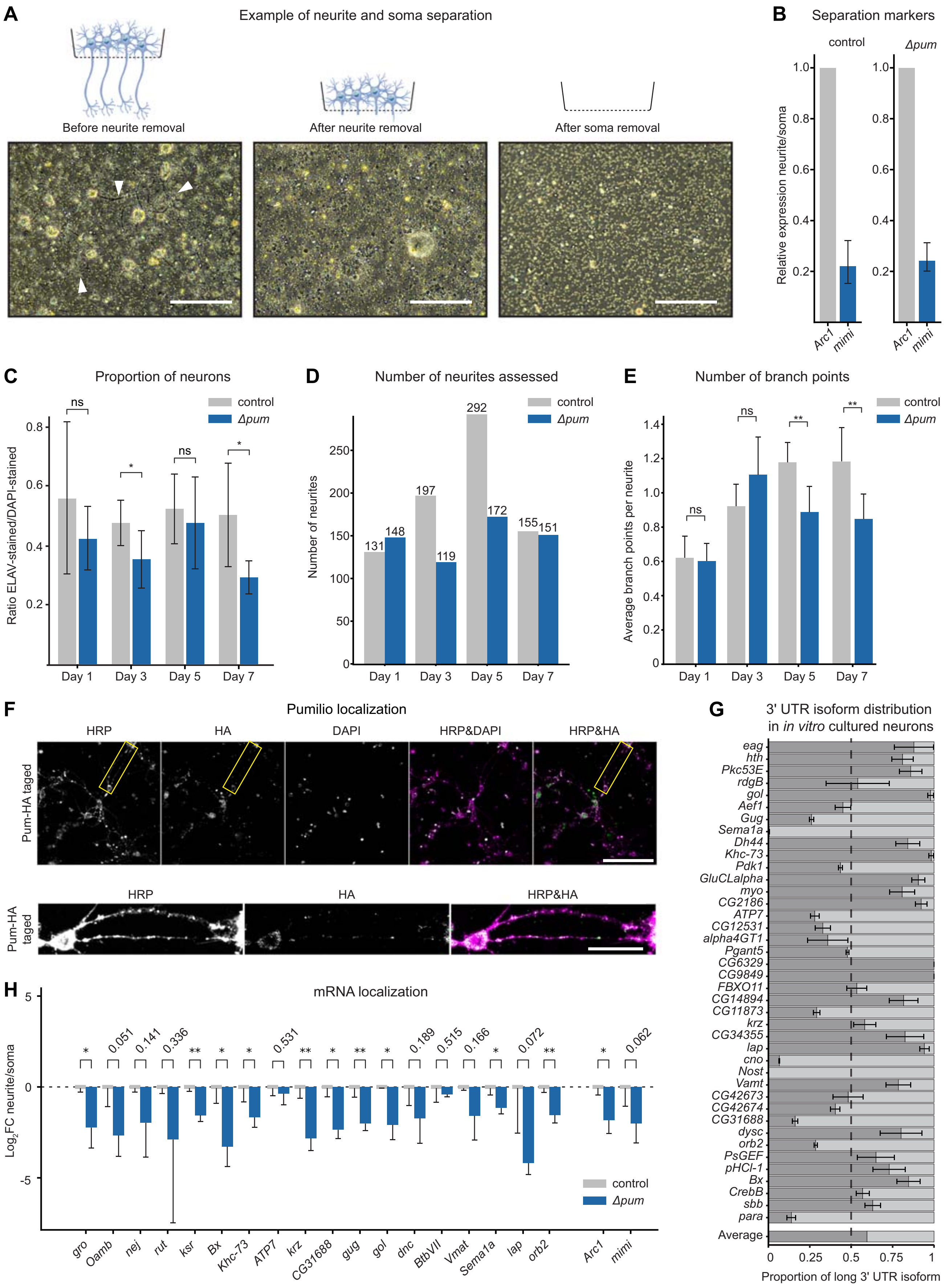
A
Example of neurite and soma separation
Before neurite removal
After neurite removal
After soma removal
B
Separation markers
control
Δpum
Relative expression neurite/soma
Arc1
mimi
C
Proportion of neurons
Ratio ELAV-stained/DAPI-stained
D
Number of neurites assessed
Number of neurites
131
148
197
119
292
172
155
151
E
Number of branch points
Average branch points per neurite
Day 1
Day 3
Day 5
Day 7
F
Pumilio localization
HRP
HA
DAPI
HRP&DAPI
HRP&HA
Pum-HA taged
G
3' UTR isoform distribution
in in vitro cultured neurons
Average
Proportion of long 3' UTR isoform
H
mRNA localization
Log2FC neurite/soma
control
Δpum

Figure EV5. Impaired neurite outgrowth, mRNA delocalization, and synaptic protein overexpression in neurons of *Δpum* flies.

(**A**) Light micrographs of Drosophila primary neuronal cells cultured on a microporous membrane for separation of neurite and soma compartments. Before removal, neurites are visible as dark protrusions (white arrowheads) below the cell bodies. After soma removal, only cell debris and micropores are visible on the membrane. The three images represent three distinct cultures at 7 days in vitro. Scale bars: 200 µm. (**B**) Assessment of soma/neurite separation by RT-qPCR quantification of two RNAs well-known to localize to distinct neuronal compartments (neurite/synapse and soma for *Arc1* and *mimi*, respectively). Shown is the RNA expression in neurites relative to cell bodies, in 7 days in vitro cultured neurons of control (genotype: w^{1118}) and *Δpum* (genotype: pum^{ET7}/pum^{ET9}) flies. For each gene, RNA levels were normalized pairwise, first to soma, and second to *Arc1*. Error bars represent the mean ± SD of three biological replicates for each genotype. (**C**) Quantification of the proportion of neurons in primary cultures from control (w^{1118}) and *Δpum* (pum^{ET7}/pum^{ET9}) dissected larval brains at the indicated days in vitro. The number of cells with ELAV staining (a marker of neuronal nuclei) was counted relative to the total number of cells with DAPI staining. Error bars represent the mean ± SD of at least 175 DAPI stained cells for each genotype and time point. *$p < 0.05$, p(3DIV control vs. 3DIV *Δpum*) = 0.03, p(7DIV control vs. 7DIV *Δpum*) = 0.04 (one-tailed Student's t-test). ns, not significant. Total number of cells quantified $n = 2100$. (**D**) Number of neurites assessed in each genotype and time point for the quantifications shown in Figs. 5C and EV5E. Total number of neurites quantified $n = 1365$. (**E**) Quantification of average number of branch points per neurite in cultured neurons of control (w^{1118}) and *Δpum* (pum^{ET7}/pum^{ET9}) flies at the indicated days after plating. Error bars represent the mean ± SD of at least 119 neurites for each genotype and time point. **$p < 0.01$; p(5DIV control vs. 5DIV *Δpum*) = 0.003, p(7DIV control vs. 7DIV *Δpum*) = 0.008 (two-tailed Student's t-test). ns, not significant. Total number of neurites quantified $n = 1365$. (**F**) Confocal imaging of C-terminally Flag-HA tagged flies (pum^{Flag}) flies at 7 days in vitro. HRP marks neuronal membranes. In merged images: HRP (magenta), HA (green) and DAPI (white). Yellow rectangles demarcate the region shown magnified in the lower panels. Scale bars: 50 µm (upper panel), 10 µm (lower panel). (**G**) Total RNA-seq quantification of long (dark gray) and short (light gray) 3′ UTR isoforms in seven days in vitro cultured neurons of control (w^{1118}) flies. 40 genes displaying shorter 3′ UTR isoforms in synaptosomes (longer 3′ UTR isoforms in input) from Fig. 3E are shown (3 genes not detected). Error bars represent the mean ± SD of seven biological replicates. (**H**) RT-qPCR quantification of the indicated transcripts in neurites relative to cell bodies, in 7 days in vitro cultured neurons of control (w^{1118}) and *Δpum* (pum^{ET7}/pum^{ET9}) flies. For each gene, RNA levels were normalized to those in control flies (in which $\log_2$ fold change = 0). Error bars represent the mean ± SD of three biological replicates for each genotype. *p*-values are indicated; **$p < 0.01$, *$p < 0.05$; p(*gro* control vs. *gro Δpum*) = 0.02, p(*ksr* control vs. *ksr Δpum*) = 0.004, p(*Bx* control vs. *Bx Δpum*) = 0.012, p(*Khc-73* control vs. *Khc-73 Δpum*) = 0.045, p(*krz* control vs. *krz Δpum*) = 0.002, p(*CG31688* control vs. *CG31688 Δpum*) = 0.02, p(*gug* control vs. *gug Δpum*) = 0.004, p(*gro* control vs. *gro Δpum*) = 2.166e−4, p(*gol* control vs. *gol Δpum*) = 0.047, p(*gro* control vs. *gro Δpum*) = 2.166e−4, p(*Sema1a* control vs. *Sema1a Δpum*) = 0.025, p(*orb2* control vs. *orb2 Δpum*) = 0.007, p(*Arc1* control vs. *Arc1 Δpum*) =0.015 (two-tailed Student's t-test).

 © The Author(s)
